# Alum an Emetic in Poisoning with Opium

**Published:** 1853-12

**Authors:** Charles D. Meigs


### *Alum an Emetic in Poisoning with Opium.*

BY CHARLES D. MEIGS, M. D.

Dr. Meigs was called on Saturday the 20th inst., at 2½ o'clock P. M., to see a young gentleman, between 26 and 28 years of age, who, after a drinking frolic, had swallowed, two hours previously, one ounce of powdered opium with the view of destroying his life. A physician of his acquaintance, who happened to be present when he swallowed the poison, immediately sent for thirty grains of sulphate of zinc, and gave it to him, but without its producing the slightest effort at vomiting. When Dr. Meigs saw the patient he was in a somnolent condition. One ounce of powdered alum was immediately procured, and one half of it, mixed with a little syrup, was given to the patient, and followed in a few minutes by two or three tumblers of warm water, when copious vomiting ensued, by which a free discharge of the contents of the stomach was induced. After the patient had rested for a short period, the remainder of the alum, followed by two tumblers of warm water, was given, which caused renewed and free vomiting, by which Dr. Meigs presumes the opium was entirely thrown off. The patient continued still to be very sleepy, but entirely recovered from the effects of his rash act. Dr. M. considered this case worth relating, as evincing the very efficient emetic powers of the alum.—*Trans. Phil. Col. Phys.*

